# Host Components Contributing to Respiratory Syncytial Virus Pathogenesis

**DOI:** 10.3389/fimmu.2019.02152

**Published:** 2019-09-12

**Authors:** Jonatan J. Carvajal, Andrea M. Avellaneda, Camila Salazar-Ardiles, Jorge E. Maya, Alexis M. Kalergis, Margarita K. Lay

**Affiliations:** ^1^Departamento de Biotecnología, Facultad de Ciencias del Mar y Recursos Biológicos, Universidad de Antofagasta, Antofagasta, Chile; ^2^Millennium Institute on Immunology and Immunotherapy, Departamento de Genética Molecular y Microbiología, Facultad de Ciencias Biológicas, Pontificia Universidad de Chile, Santiago, Chile; ^3^Departamento de Endocrinología, Facultad de Medicina, Pontificia Universidad Católica de Chile, Santiago, Chile

**Keywords:** RSV, pathogenesis, innate and adaptive immune response, host factors, disease

## Abstract

Respiratory syncytial virus (RSV) is the most prevalent viral etiological agent of acute respiratory tract infection. Although RSV affects people of all ages, the disease is more severe in infants and causes significant morbidity and hospitalization in young children and in the elderly. Host factors, including an immature immune system in infants, low lymphocyte levels in patients under 5 years old, and low levels of RSV-specific neutralizing antibodies in the blood of adults over 65 years of age, can explain the high susceptibility to RSV infection in these populations. Other host factors that correlate with severe RSV disease include high concentrations of proinflammatory cytokines such as interleukins (IL)-6, IL-8, tumor necrosis factor (TNF)-α, and thymic stromal lymphopoitein (TSLP), which are produced in the respiratory tract of RSV-infected individuals, accompanied by a strong neutrophil response. In addition, data from studies of RSV infections in humans and in animal models revealed that this virus suppresses adaptive immune responses that could eliminate it from the respiratory tract. Here, we examine host factors that contribute to RSV pathogenesis based on an exhaustive review of *in vitro* infection in humans and in animal models to provide insights into the design of vaccines and therapeutic tools that could prevent diseases caused by RSV.

## Introduction

Respiratory syncytial virus (RSV) is the main viral etiological agent that produces lower respiratory tract infections (LRTI) and is the primary cause of hospitalization due to respiratory diseases in infants (1, 2). RSV infection may lead to bronchiolitis and pneumonia and has been implicated in the development of recurrent wheezing and asthma (3). Milder RSV manifestations include rhinorrhea, cough, congestion, low-grade fever, reduced appetite, and respiratory distress (4). Recent reports of other pulmonary manifestations, such as encephalitis, cardiopathy, and hepatitis, suggest that RSV has a versatile ability to infect tissues of the respiratory tract (5).

RSV is highly infectious and easily spread in hospitals, homes, and nurseries, despite being less cytopathic and less invasive than influenza A virus. Worldwide, RSV affects more than 70% of infants in the first year of life, and nearly 100% of children by 2 years of age (6). The estimated rate of hospitalization due to RSV is 3.4 million/year and between 66,000 and 239,000 deaths occur around the world in children under 5 years of age who have suffered LRTI caused by RSV (7, 8). During the year 2000 in the U.S., there were approximately 86,000 RSV-associated hospitalizations, 402,000 emergency room visits, 1.7 million office visits, and 236,000 outpatient hospital visits, at an estimated cost of US $652 million (9). Interestingly, the rate of hospitalization for primary RSV infection in Alaska is approximately 0.5%, but can vary by situation, with ethnic group susceptibility as high as 25% (10). In contrast, an estimated of 33.1 million cases of RSV LRTI was reported in children under 5 years of age in 2015. Half of the global RSV burden was contributed by cases in India (7,013,468), China (2,581,262), Nigeria (1,728,622), Pakistan (1,575,051), and Indonesia (1,245,185) (11). RSV is also recognized as a major threat to older adults (>64) (12). Epidemiological evidence indicates that the impact of RSV on these patients may be similar to non-pandemic influenza (12).

Scientific evidence has shown that after the resolution of respiratory diseases associated with RSV infection, the virus interferes with the establishment of immunological memory, which leads to recurrent reinfections (13). Indeed, around 36% of individuals can be reinfected with RSV, at least once, during the winter season (13). These reinfections could result when an initial encounter with RSV fails to initiate adequate humoral and cellular immune responses to generate protective memory lymphocytes (13, 14).

RSV was first isolated in 1956, from throat samples in a colony of chimpanzees that had symptoms such as coughing, sneezing, and purulent nasal discharge (15, 16). These symptoms were quickly observed in other monkeys of the colony, indicating that the pathogen responsible for the disease was highly contagious. Originally, the pathogen was called chimp coryza agent (16). Later, in 1957, a similar viral agent was isolated from the throats of babies who had severe respiratory diseases (17). Interestingly, the isolated pathogen induced syncytia formation that was shown later to be caused by the viral fusion (F) protein (18, 19). Since then, this pathogen was renamed as RSV.

This respiratory virus was recently classified in the *Pneumoviridae* family, *Orthopneumovirus* genus (20). Specifically, RSV is an enveloped, negative sense, single stranded RNA virus with a non-segmented 15.2 kb genome, containing ten genes: non-structural proteins (NS)1, NS2, nucleoprotein (N), phosphoprotein (P), matrix (M), small hydrophobic (SH), fusion (F), glycoprotein (G), M2 and large polymerase (L) (from the 3′ to 5′ end) that encode eleven proteins (21). The M2 gene contains two open reading frames that slightly overlap and encode the M2-1 and M2-2 proteins (22). Further, the F, G, and SH proteins are found on the viral surface, whereas the N, P, L, M, and M2 proteins are located underneath the viral envelope (21, 23). The F protein is essential for union and entry of the virus into the host (24, 25). F and G are the only RSV proteins that induce neutralizing antibodies (26).

A growing concern is that severe RSV infection at an early age, may adversely affect pulmonary development and lead to long-term respiratory disorders. Thus, the development of new treatment strategies to prevent RSV infections is a priority of the World Health Organization (27). To design effective therapeutic tools that thwart viral infection, we need to understand host factors that influence RSV pathogenesis. In this review, we describe mechanisms of RSV pathogenesis, as well as host factors and immune responses that contribute to disease severity caused by this important respiratory virus.

## RSV Pathogenesis

RSV transmission occurs via air, by contact with epithelium of the nostrils, mouth, or eyes of RSV-infected individuals, or by contact with a surface contaminated with the virus (28). RSV can survive for prolonged periods on the surface of furniture (7 h), skin (30 min), fabrics (2 h), and gloves (5 h), which facilitates its spread (29, 30). With an incubation time of 3–8 days, RSV can infect the lower respiratory tract producing bronchiolitis (inflammation of bronchioles in the small airways) or pneumonia (inflammation of the alveolar spaces in the small airways). In children, pneumonia caused by RSV manifests with fever, chest pain, wheezing, nausea, chills and other respiratory difficulties (31, 32). Likewise, bronchiolitis caused by RSV is characterized by wheezing, dyspnea, tachypnea, fatigue, fever, and cough (33). Because these diseases could be fatal, infants with severe RSV symptoms are hospitalized to receive necessary health care.

Once RSV enters the nostrils or mouth, it begins to infect airway epithelial cells (AECs) of the upper respiratory system (34–36), moving down to the lower respiratory system, and reaching the bronchioles where viral replication is more effective, as observed in both mouse and infant respiratory tissues (37, 38). Specifically, ciliated cells in the bronchial epithelia and type 1 pneumocytes in the alveolus, are the main cells targeted by RSV infection (39–42). RSV has also been reported to infect intraepithelial dendritic cells (DCs) and basal epithelial cells of the conductive airways, using *in vitro* cultures (41). Thus, RSV has a wide range of cellular reservoirs in the respiratory tract that perpetuate its pathogenesis in the human host.

An *in vitro* AEC model was used to show that RSV infection is concentrated in groups of non-continuous cells or small groups of ciliated apical cells located in the epithelium of large airways (40). As this infection progresses, RSV induces sloughing and shedding of specific apical AECs, loss of ciliation, as well as sporadic syncytium formation and mucus hypersecretion, which could lead to formation of thick plugs in the bronchiolar lumen *in vivo* (40, 43, 44). RSV has also been shown to cause detachment of apical AECs *in vivo*, which exposes nociceptive nerve fibers and produces a cough reflex (37).

Well-differentiated primary pediatric bronchial epithelial cells (WD-PBEC) provide a suitable *in vitro* model to study RSV infection (45, 46). WD-PBECs consist of polarized pseudostratified multilayered epithelium containing ciliated, goblet and basal cells and intact tight junctions. Hence, this *in vitro* model imitates the physiological, functional, and morphological environment of the respiratory tract (47).

RSV does not cause the massive airway epithelium destruction observed in post-mortem lung samples from RSV-infected patients in the WD-PBEC *in vitro* model (39, 44, 45). The latter *in vitro* studies demonstrated that the cell monolayer remains intact, even when most of ciliated cells were infected with RSV. Other studies confirmed these results and demonstrated that peak RSV infection in ciliated cells occurs at day 4 post infection (p.i.) and decreases significantly by day 8 p.i. (48), suggesting that in the absence of immune-cell mediated mechanisms, RSV-infected ciliated cells can be cleared from the epithelium between days 4 and 8 p.i. Detachment and apoptosis of ciliated cells in the epithelium of apical airways has also been observed in WD-PBEC cells, which agrees with the results of histopathological studies of infants with fatal RSV, where caspase-3 activity was detected in bronchiolar epithelial cells (49). Using an *in vitro* model, Liesman et al. (48) also found that RSV-infected ciliated cells die when they detach from the epithelium. RSV-infected AECs that have sloughed from the airway epithelium are thought to obstruct the lower airways in RSV-infected individuals, as observed in hospitalized infants (48). RSV NS2 was identified as the viral protein that causes rounding and sloughing of infected ciliated cells by using RSV gene deletion mutants and gain-of-function experiments with recombinant RSV NS2-expressing parainfluenza virus 3 (PIV3-NS2) (48).

An *in vivo* model was also used to show that RSV pathogenesis is characterized by excessive mucus secretion (48). However, RSV does not infect goblet cells, nor does it induce them to secrete mucus. Rather, it infects basal cells of the airway epithelium, which differentiate into mucus-secreting cells, as shown in an *in vitro* culture model (48). Thus, RSV indirectly induces mucus in the bronchial lumen by stimulating goblet cell proliferation, consistent with the presence of goblet cell hyperplasia in lungs of fatal RSV infection cases (49). Therefore, RSV infection in the respiratory tract induces the production of mucus plugs and detached ciliated AECs. Additionally, RSV drastically reduces mucociliary transport (MCT), a unidirectional movement of the airway epithelium that mobilizes mucus plugging out from the airways within 5 days. Thus, these plugs accumulate in the bronchial lumen, leading to the pathogenesis of this viral agent (48). Consequently, RSV infection produces epithelial airway necrosis, submucosal edema, and occlusion of the bronchial lumen (37, 39–41).

RSV was also recently shown to induce production of thymic stromal lymphopoietin (TSLP) and interleukin (IL)-33 (50–54), which are cytokines that play important roles in the development of allergic asthma. *In vivo* and *in vitro* models were used to show that production of these cytokines has important repercussions on RSV pathogenesis. Indeed, upon RSV infection in the lungs, TSLP and IL-33 secretion create an inflammatory environment that directly or indirectly increases mucus secretion, eosinophil and neutrophil numbers, and levels of the T helper (Th) 2 cytokine interleukins IL-5 and IL-13 (55–59), as discussed in more detail in the section below. Concurrently, a decrease in the total CD4+ and CD8+ T-lymphocyte numbers is also observed in RSV-infected individuals (60, 61).

**Figure 1:**
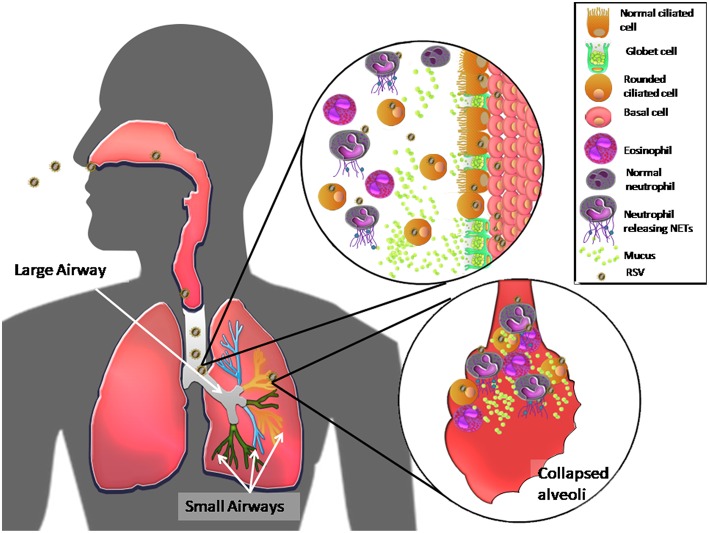
Model of RSV pathogenesis in the human respiratory tract. Once RSV enters the upper respiratory tract, the virus primarily infects ciliated cells in the large airways. The viral NS2 protein induces cell rounding, extrusion, and detachment from the apical zone of the airway epithelium through, generating an accumulation of these cells in the lumen of the airway ducts. RSV also induces proliferation of goblet cells, via infection of basal cells, which differentiate, causing high mucus production and recruitment of eosinophils and massive numbers of neutrophils in the airway ducts. The latter immune cells release NETs in response to RSV infection. All of these components accumulate and move to the distal airways, leading to bronchial obstruction of the narrow ducts, and collapse of alveoli, causing acute inflammation and pathology in the lungs.

Together, these results suggest that RSV, as a result of its NS2 protein functions, primarily infects ciliated cells in the large airways, inducing their extrusion and cell shedding into large airways. RSV also induces mucus production by causing goblet cell proliferation and proinflammatory cytokine production. Mucus accumulates in the narrow-diameter bronchiolar airway lumen due to RSV inhibition of MCT, which, in turn, is thought to cause acute obstruction in the distal airways. RSV infection of ciliated cells also induces TSLP and IL-33 release, which indirectly induces eosinophil and neutrophil recruitment to the lung. In other respiratory viral infections (62), *in vivo* presence of the latter immune cells correlates with the presence neutrophil extracellular traps (NETs), which are web-like networks of neutrophil DNA covered with histones and cytotoxic microbicidal proteins that trap and eliminate different pathogens (62). The release of NETs by neutrophils has been observed *in vitro* (62). Thus, upon RSV infection, recruited neutrophils and tamponades produce NETs, composed of mucus and dead ciliated cells that appear to exacerbate obstruction of the host upper and lower airways (62, 63). Figure 1 shows a current RSV pathogenesis model.

### RSV Pathogenesis in Infants

RSV is a highly infectious virus, especially in infants and young children. At early ages, most primary RSV infections cause LRTI, resulting in hospitalization for an estimated 2–3% of infected infants. LRTI in infants and young children can result in respiratory diseases including bronchiolitis, pneumonia, wheezing, and even respiratory failure, which likely ends, unfortunately, in death. RSV is considered the second most-likely single pathogen to cause death in children <1 year of age (8). Possible host and virulence factors that determine the outcome of LRTI in infants upon RSV infection will be discussed below.

### RSV Pathogenesis in Adults and the Elderly

RSV pathogenesis in adults and the elderly differs from that in infants, displaying symptoms similar to those caused by influenza virus, typically including mild fever, runny nose, nasal congestion, cough, dyspnea, and wheezing (64, 65). A study of adults between 24 and 95 years of age who had been exposed to RSV, showed detectable virus for 10 to 13 days in nasal secretions that in some cases lasted ≥20 days. Levels of RSV viral RNA in sputum were slightly higher than nasal titers, suggesting that viral replication also occurs in the lower airways in adults (12, 66).

In adults, it is required that a diagnosis of RSV infection based on laboratory diagnostic tests be confirmed due to the similarity of RSV symptoms with other viral and bacterial agents that cause acute respiratory tract infection. Reverse transcriptase-polymerase chain reaction (RT-PCR) is the reference diagnostic method for RSV detection and is specifically recommended for use in adults because its analytic and clinical sensitivities are superior to those of other diagnostic methods (67, 68). However, a low percentage of clinical laboratories currently use RT-PCR to identify RSV because of its associated costs, specialized equipment, and expertise required (69). Consequently, most RSV disease in adults is not diagnosed early (70, 71). The absence of an easily administered and effective antiviral and a commercially available vaccine has led to a high rate of severe RSV disease in the elderly (72). Indeed, RSV infection rates in nursing homes are ~5–10% per year, with significant rates of pneumonia (10–20%) and death (2–5%) (73). Data collected in the U.S.A. over 9 years of surveillance indicated that RSV infection causes approximately 10,000 deaths per year in people over 64 years of age. In addition, some *in vivo* studies conducted in this risk group reported high levels of IL-6 and macrophage inflammatory proteins 1 alpha (MIP-1α) upon RSV infection, which directly correlates with the severity of patient disease (73, 74). Although the protective or pathological roles of cellular immunity in adults is still unknown, *in vivo* studies indicate that there is decreased production of interferon (IFN)-γ and both CD8+ and CD4+ memory T cells with age, which could influence the severity of RSV disease (75–78). These immune host factors for RSV susceptibility will be discussed more in detail in the next section.

## Host Components Contributing to RSV Pathogenesis

Several host factors affect RSV pathogenesis and increase the risk of developing severe RSV disease including young age (<6 months), premature delivery (<35 weeks of gestation) (79, 80), malnutrition (81), gender, low titer RSV-specific serum antibodies, and fragile old age (82). Suffering from severe or chronic diseases, including allogeneic bone marrow transplants (83), congenital heart defects (84), chronic lung disease including cystic fibrosis (85), and nervous system and muscle diseases (86, 87) increases the risk of severe RSV disease in older adults and in babies (88). Babies born with Downs syndrome and cerebral palsy have also been shown to have a higher risk of hospital admission with RSV bronchiolitis, although more research is needed to better explain the mechanism behind this risk factor for RSV infection (89). Host conditions that contribute specifically to pathogenesis of severe LRTI caused by RSV (90) include immune system immaturity and immunologic impairment disorders, incomplete development of the respiratory tract, hyperreactivity of the airways, and pulmonary congestion. Epidemiological studies have also established that primary infection at an early age plays a central role in RSV disease severity (91).

### Early Age

Inability of the infant immune system to efficiently respond after RSV infection is due, at least in part, to failure of innate antiviral immune responses (92). Studies in infants, including fatal cases, found that after RSV infection, respiratory epithelial cells release chemokines and cytokines that are known to recruit immune cells to the site of infection, such as leukocytes, neutrophils, monocytes, natural killer (NK) cells, macrophages, eosinophils, basophils, and DCs, which contribute to lung inflammation (92). Low expression of Toll-like receptor (TLR) 4 in the neutrophils of infants (93) could also contribute to development of more serious RSV disease caused in this population (93). The status of innate immune cells in the host may also contribute to RSV pathogenesis. In fact, *in vivo* assays of hematopoietic cells found that they are permissive for RSV infection and can serve as an RSV reservoir (93). For example, DCs play a central role in configuring the immune response to, and disease outcome of, RSV infection (94).

Plasmacytoid (p)DCs are thought to be key players in the immune response to different viruses, due to their ability to produce large amounts of type I IFN (IFN-α and IFN-β) (94). These cells are known to be important in controlling RSV infection in mouse lungs (94, 95). RSV-induced IFN-α, mainly produced by pDCs, is significantly lower in term infants and young children (<5 years of age), than in adults, suggesting that human pDCs have a limited function in early life that could partially explain the severity of RSV disease in infants and young children (96). Further studies are required to fully elucidate the role of pDCs in RSV disease.

Neonatal susceptibility to RSV is intrinsically linked to immunological characteristics of the young pulmonary mucosa. To better understand immune responses to RSV infection in infants, a mouse model of neonatal infection was developed in BALB/c mice (96). Mice infected with RSV within 7 days of birth developed an asthma-like pathology and when these mice were reinfected as adults, they underwent weight loss, airway hyperresponsiveness, mucus hypersecretion, Th2 immune responses, and airway remodeling (95, 96). These results suggest that primary RSV infection at an early age in neonatal mice influences the clinical outcome of RSV re-exposure in adults.

### Gender

Gender is another host factor that can affect RSV disease susceptibility. For example, illness caused by RSV infection is more severe in male infants because their airways have a smaller diameter than those of female infants (97, 98). Thus, male infants are more likely than females to have an acute bronchial obstruction upon RSV infection.

### Hypersensitivity

Hypersensitivity, such as allergic reactions, is an exaggerated immune response to an antigen. There is a strong epidemiological correlation between severe RSV infection in early life and asthma development later in life (27, 99, 100). Some evidence in infants shows an association between genetic predisposition to asthma and disease severity following RSV infection. During RSV infection of infants, a large amount of specific immunoglobulin (Ig) E is produced (101, 102) and increased sIgE levels correlate with greater severity of RSV infection such as wheezing in babies and asthma in children (101, 103). Similarly, RSV-infected asthmatic patients had higher anti-RSV IgE antibody titers than did non-asthmatic individuals (104), suggesting that RSV infection differs between asthmatic and non-asthmatic individuals.

Atopic hypersensitivity also correlates with severe RSV disease (105). Specifically, 32% of children hospitalized with RSV infection developed atopic sensitization, while only 9% of those who were not hospitalized due to RSV infection developed atopic sensitization after 3 years (*p* = 0.002). In a follow-up study, 34% of patients hospitalized due to RSV infection developed atopic hypersensitivity to allergenic agents by 7 years of age compared to only 15% of those who had not been hospitalized due to RSV infection (106).

Cytokines such as IL-3, IL-4, IL-10, and IL-13 in the lower respiratory tract of infants with RSV bronchiolitis (107) are known to exacerbate allergic processes (108–110). The presence of some of these cytokines correlates with severe RSV infection. Specifically, Bertrand and Lay (107) reported a direct correlation between the number of days hospitalized due to RSV infection and high IL-4 levels (Pearson correlation: *r* = 0.52, *p* = 0.05) (107). They also found a direct correlation between high levels of IL-12p40 and IL-3, and development of recurrent wheezing later in life upon RSV infection (Pearson correlation: *r* = 0.68, *p* = 0.0071, *r* = 0.71, *p* = 0.0058, respectively) (107). These two cytokines were elevated in infants who developed asthma later in life. Interestingly, IL-3 is known to be involved in mast cell infiltration into the airways as well as increased basophil production (111). The same study found elevated expression of IL-33 mRNA in nasopharyngeal aspirates from RSV-infected patients with a family history of atopy (107), suggesting that genetic predisposition of the host promotes Th2 responses and allergic inflammation after RSV infection, similar to previous reports of sensitization to allergens (112–115).

### Genetic Factors

Genetic predisposition of the host could affect RSV disease severity (89, 116) and recent studies have proposed that genetic predisposition to asthma could also predispose to severe RSV disease (117, 118). Specifically, a clinical study by Thomsen et al. (117) found that hospitalizations due to RSV and asthma were directly correlated (*r* = 0.43), and that genetic determinants of the two disorders overlap precisely. The same study analyzed the correlation between hospitalization due to RSV and asthma, showing a model, by which asthma “causes” hospitalization due to RSV. This model was adjusted to data significantly better (*P* = 0.39) than one by which RSV hospitalization “causes” asthma (*P* < 0.001). In support of this model, recent studies also showed that asthma increases the risk of RSV hospitalization by 3-fold in a time-independent manner (119, 120). However, the exact mechanisms by which severe RSV infection interacts with asthma inheritance factors at the onset of childhood RSV infection still need to be elucidated.

Other genetic factors associated with severity of RSV disease are two single nucleotide polymorphisms (SNPs) that encode Asp299Gly and Thr399Ile substitutions in the TLR4 ectodomain, which were previously associated with TLR4 and are known to regulate innate and adaptive immune responses by recognizing pathogen-associated molecular patterns (PAMPs) (121). Both SNPs are related to increased severity of RSV infection in premature babies, with 89.5 and 87.6% of heterozygous cases for Asp299Gly and Thr399Ile polymorphisms, respectively (121). These results suggest that heterozygosity of these two TLR4 SNPs is strongly associated with symptomatic RSV disease in high-risk infants, supporting a dual role for TLR4 SNPs in prematurity and increased susceptibility to RSV (121). *In vitro* studies also showed that these SNPs were associated with a decreased response to lipopolysaccharide (LPS) and to purified RSV F protein that activates cells through TLR4 (122). Thus, these mutations may delay and/or attenuate triggering of the innate immune response to RSV (96).

Another genetic factor that has been reported to be involved in RSV disease is the CC genotype of CD14 (−550 C/T), which is associated with development of RSV bronchiolitis in Japanese populations (123). This study found that CD14 (−550 C/T) is associated with higher serum levels of soluble (s) CD14 in Japanese neonates and children and directly correlates with development of bronchiolitis upon RSV infection. A possible explanation of these associations is that high levels of sCD14, a soluble form of the glycosyl phosphatidylinositol–anchored membrane protein (124), may bind to available LPS and transfer it to membranous CD14, thereby stimulating production of proinflammatory cytokines such as tumor necrosis factor–α (TNF-α) (125–127), which is known to enhance RSV-induced disease (128). RSV bronchiolitis could be triggered by RSV infection and an inflammatory environment caused by high sCD14 levels in the blood.

Although these are very important host factors, several authors have suggested that Th2 cytokine genes such as IL-4, IL-13, and IL-5 contribute to asthma severity. A study of single-strand conformation polymorphism in these genes, which are grouped on chromosome 5, identified point mutations at IL-3 position−68, IL-4 position−590 and IL-9 position−351 (129). The IL-4 promoter polymorphism is associated with increased total serum IgE, which is of special interest, since this group of cytokines is involved in asthma development and could be influenced during and after RSV infection (130). These genes are also associated with exacerbating and perpetuating asthma during RSV infection (129).

Four SNPs of interest have also been shown to be associated with RSV disease severity at allele and at genotype levels. Specifically, a SNP in the vitamin D receptor gene (rs10735810, *P* = 0.0017) (131) has been linked to increased susceptibility to RSV infection (132). In addition, the synthetic nitric oxide 2 (NOS2A) gene (rs1060826; *P* = 0.0031) (131) has been associated with increased chronic respiratory morbidity and reduced lung function in infants who had LRTI caused by RSV (133). Further, the Jun protooncogene product, a subunit of the AP-1 transcription factor (JUN) (rs11688; *P* = .0093) (131) and interferon alpha 5 (IFNA5) gene (rs10757212; *P* = 0.0093) (131) are involved in innate immunity and contribute to the susceptibility to and duration of RSV infection (134, 135).

The olfactory receptor (OR13C5) gene is also involved in RSV pathogenicity, since the olfactory nerve connects the nasal cavity with the central nervous system and thus could be used as a shortcut by RSV (136). This could explain neurological symptoms produced by RSV such as encephalitis, apnea, or seizures, that occur in at least 2% of RSV-infected people (137, 138), and likely cause serious and permanent neurological *sequelae* (139). SNPs in human leukocyte antigen (HLA) HLA-DQA1 and in HLA-DPB1 genes have been associated with the development of bronchiolitis and several types of asthma (140–142). One of the most important SNPs is located in the mucin 4 (MUC4) gene, where three SNPs have been identified: rs201623571 (*P* = 3.55 × 10^−10^, OR0.10), rs529417345 (*P* = 9.40 × 10^−10^, OR = 0.03) and rs548345415 (*P* = 9.40 × 10^−10^, OR = 0.03). These SNPs in MUC4 diminish mucin in the airways upon RSV infection, which can increase the severity of RSV disease (143, 144).

In a severe RSV infection, loss of function (LOF) variants associated with the innate immune response, such as helicase C domain 1 (IFIH1) and other IFN pathway genes become very important (145). IFIH1 encodes a RIG-I-like cytoplasmic sensor that detects viral RNA by interacting with its C-terminal regulatory domain (CTD) and helicase domain with long dsRNA molecules. This ATP-dependent reaction polymerizes IFIH1 molecules into a filament, and assembles IFIH1 caspase activation recruitment domains (CARDs), which in turn induce IFN-β expression and activate antiviral genes (146). IFIH1 has been shown to effectively restrict RSV replication. Specifically, Asgari et al. (145) showed that three IFIH1 LOF variants increase the susceptibility and duration of RSV infection. One of those was a “rare splice” variant rs35732034 (145) that changed the reading frame and produced an early stop codon, whose protein (IFIH1-Δ14) lacks a CTD. IFIH1-Δ14 cannot bind viral dsRNA and has thus lost its main function. The second variant is a “cracking” variant rs35337543 (145), whose protein (IFIH1-Δ8) removes amino acids at the end of the helicase 1 domain and in the helicase 1 and 2 binding site without changing the reading frame. Finally, the “prolonged gain” variant rs35744605 protein (IFIH1-ΔCTD) lacks amino acids at the C-terminus (145). These three variants are unable to induce IFN-β, having lower stability than the normal protein and lacking the characteristic ATPase activity required to polymerize and activate IFH1. These IFIH1 gene variants impair normal function of the viral sensor protein, thus restricting RSV infection (145).

Other genes that contribute to RSV disease severity, duration, and susceptibility include SFPA/D, IL-8, IL-4, and IL4RA, which exacerbate bronchiolitis caused by RSV (147–150); as well as IL-10 and IL-13 genes. Patients with mutations in IL-10 and IL-13 have required mechanical ventilation upon RSV infection (151, 152). In addition, SNPs in genes related to the innate immune response such as IFNA13 (rs643070), IFNAR2 (rs7279064), signal transducer and activator of transcription (STAT) 2 (rs11575234), IL27 (rs181206), Nuclear Factor Kappa B Inhibitor Alpha (rs22333409), C3 (rs22302021), IL1RN (rs315952), and TLR5 (rs5744174), have been associated with failure of the antiviral response against RSV (131, 153). Moreover, the ADAM33 and transforming growth factor beta receptor 1 (TGFBR1) genes participate in respiratory tract remodeling and increase RSV disease severity by favoring viral replication (153–155).

### Malnutrition

Vitamin D plays a major role in innate immunity and influences lung function of asthmatic patients (156). In its active form, vitamin D 25-dihydroxyvitamin D [25(OH)D] helps to modulate inflammatory processes (157–160), promote Treg cell development (160), and acts as an antiviral agent (161). The concentration of 25(OH)D in the blood has been associated with the risk of contracting severe respiratory infection or exacerbating asthma in children and adults (162–164). The risk of contracting a severe respiratory infection decreases by 7% for every 10 nmol/L of 25(OH)D in adults (165). Some studies indicate that the risk rises in children and in infants when 25(OH)D concentrations fall below 75 nmol/L, making these children more vulnerable to bacterial and viral lung infections (166–168). In recent studies, low levels of vitamin D in cord blood of healthy neonates was associated with an increased risk of severe RSV LRTI in the first year of life (169, 170), suggesting that a low intake of this vitamin by the mother during pregnancy can impact RSV disease severity in infants.

RSV reinfection can occur throughout life, causing winter/early spring epidemics in temperate regions, but synchronization of RSV activity can vary widely depending on geographical location. It should be noted that different RSV strains circulate rapidly throughout the world (171). Environmental factors (temperature and humidity), including those that affect lung function (e.g., smoking at home), external conditions that increase exposure to RSV infection (e.g., daycare, hospitalization, multiple siblings), and lack of lactation, are factors that may indirectly influence RSV disease severity (28).

The host response to RSV infection has largely been studied in infants with comorbidities, but not in healthy infants or in the elderly. Although there is no vaccine or effective antiviral therapy currently, there is much effort to investigate these issues (172–174). Babies at high risk for serious RSV disease can receive passive immunoprophylaxis during an epidemic season by monthly injection of the RSV neutralizing monoclonal antibody, palivizumab (Synagis), which provides a 55% reduction in hospitalization rate associated with RSV (175).

### Premature Birth

As mentioned above, infants under 6 months of age have an increased risk of RSV infection. However, premature infants, with a gestational period of <37 weeks are even more likely to develop severe RSV bronchiolitis than full-term infants (176). One reason for this risk is the deficiency in passive immunization by maternal antibodies that are essential to defend against pathogens in the first months of life. Moreover, during gestation antibodies migrate from the mother to the fetus between 26 and 41 weeks of gestation. These antibodies include IgG1 and IgG4 that are efficiently transferred from the mother to the fetus, followed by IgG3 and IgG2 (177–180). Because premature infants with <41 weeks of gestation have not fully acquired maternal antibodies, they have an increased risk of RSV infection. Another important immunological factor in premature infants is the presence of neutrophils, which have a reduced ability to migrate to respiratory tissues than in full-term infants (181). Neutrophils in premature infants also release fewer bactericidal proteins and have decreased pathogen recognition capacities (176, 182). Premature infants also have compromised pulmonary development. One of the complications is bronchopulmonary dysplasia (BPD), which is abnormal development of lung tissue. Infants with this disease upon RSV infection, have a more severe outcome with a much higher rate of hospitalization and death (176, 183).

### Microbiome of the Airways

RSV infection in the lower respiratory tract of infants who develop severe bronchiolitis has been associated with a specific microbiota that includes a high abundance of Firmicutes, such as the genus *Streptococcus*. These patients were reported to have a low abundance of Proteobacteria, including the genera *Haemophilus* and *Moraxella* (*P* < 0.001) (183, 184). However, another study reported that children <2 years of age who were hospitalized due to RSV infection had a positive association with the presence of *H. influenzae* and *Streptococcus* and a negative association with *S. aureus* (Firmicutes phylum) abundance (185). A third study showed that nasopharyngeal aspirates of RSV-infected infants (<6 months), with different levels of disease severity, had an abundance of opportunistic organisms like *Haemophilus* and *Achromobacter*. The abundant presence of *Haemophilus* in these RSV-infected patients was associated with increased viral load and mucosal chemokine (C-X-C motif) ligand 8 (CXCL8) responses, which influence RSV disease severity (186). Further studies are needed to elucidate the role of respiratory tract microbiota in RSV disease.

### Factors Associated With Age

Aggravating factors such as chronic obstructive pulmonary disease can exacerbate RSV infection in the elderly (184, 187). Being a smoker also increases the chances of developing asthma after RSV infection and exacerbates the pathology if the individual already has asthma (188, 189). Other underlying chronic lung diseases, such as bacterial coinfection in the airways, can increase the severity of RSV infection, including death (189). An acute RSV infection can trigger an acute myocardial infarction in adult patients (189). Immune senescence combined with decreased numbers of RSV-specific neutralizing antibodies in the serum of this patient group (75, 190, 191) can have a detrimental effect on RSV infection.

### Role of Regulatory T Cells

Regulatory T cells (Tregs) are immunomodulatory cells that play a key role in tolerance, immune homeostasis, and regulating inflammatory responses by suppressing T-cell proliferation and cytokine production (192). Tregs avoid exacerbating the immune response (193–195), which can be harmful to an individual. Although, Tregs are found in newborns and adults, preterm infants have a higher number of Tregs than do full-term infants (196), while adults have fewer Tregs than do full-term infants (197). However, Tregs from adults more efficiently suppress T-cell responses than do Tregs from children (198). Conversely, Tregs from newborns are more resistant to apoptosis than are Tregs from adults (199). The frequency of activated Tregs was lower in the peripheral blood of infants infected with RSV than in age-matched controls. These results suggest that the reduced number of Tregs in RSV-infected infants precludes their ability to properly control the host inflammatory response leading to severe RSV disease in these patients (200). Further studies still are needed to understand the contribution of Tregs to RSV disease.

## Host Immune Response to RSV

### Host Innate Immune Response Against RSV

Once RSV enters the host respiratory tract, it begins to infect susceptible target cells in the respiratory epithelium. The host responds through pattern recognition receptors (PRRs) that activate early innate immune responses at the site of infection (47, 201). PRRs can detect PAMPs, including RNA viruses like RSV that infect the respiratory tract (202). These interactions induce cytokine production, including IFN, and antiviral responses (47). A majority of TLRs, RIG-I-like receptors (RLRs), nucleic acid-binding domains, and leucine-rich proteins (LRRs), are involved in antiviral defense and in increasing cytokine production during RSV infection (203, 204). Recognition of RSV by these PRRs is well-studied in humans and in adult mice, but very little is known about their role in neonates.

Early host detection of RSV occurs through three main classes of PRRs. First, TLRs activate the innate immune response via myeloid differentiation primary response 88 (MyD88) (TLRs 2, 4, 7 and 8) or via the TIR-domain-containing adapter-inducing interferon-β (TRIF) (TLR 3 and 4). Once a specific PAMP is recognized (205), RLRs such as RIG-I, melanoma differentiation-associated protein 5 (MDA5), and nucleotide-binding oligomerization domain-containing protein (NOD) 2, activate the innate signaling pathway through the adapter mitochondrial antiviral-signaling protein (MAVS) (206) and NOD-like receptors (NLRs). However, other cellular proteins, such as protein kinase R (PKR) may also recognize RSV in infected cells (207). The signal generated by PRRs activates transcription factors such as the regulatory factors NF-κB, JUN, and different IFN regulatory factors (IRFs). These factors then induce type I IFN expression, DC activation, and expression of proinflammatory cytokines and chemokines, that are produced not only by DCs but also by cells such as alveolar macrophages in the respiratory tract (208). Production of IFN types I and III is induced in this early immune response against RSV, resulting in transcription of IFN stimulating genes and production of proinflammatory mediators. RSV infection activates the inflammasome, cellular stress, and in some cases cell death (209). The role of TLRs in response to RSV infection was evaluated in TLR deficient mice. Peritoneal macrophages from C57BL/6, TLR2 KO, and TLR4 KO mice, previously induced with thioglycolate, were then stimulated with RSV. Mouse macrophages from TLR2 KO and TLR4 KO mice produced lower levels of intracellular TNF-α than did wild type mice after RSV infection. Further, macrophages from TLR2 KO mice generated the lowest TNF-α levels, suggesting that TLR2 plays an important role in proinflammatory cytokine induction after RSV infection (210). Likewise, TLR2 KO mice infected with RSV displayed altered migration of neutrophils to the lung and uncontrolled RSV replication, despite type I IFN production (211). Although TLR3 recognizes viral dsRNA, studies suggest that it may not be required for viral clearance of RSV infection, nonetheless, it is important to maintain an adequate environment in the lung. Similarly, an altered immune environment is induced, affecting the airway epithelium, without TLR7-mediated responses (212, 213).

TLR3 contributes to RSV recognition during infection, since it binds to viral RNA that is generated during replication (47). However, once the viral RNA is detected, both TLRs and RLRs provoke a signaling cascade that activates the transcription factors NF-κB, IRF, and activating transcription factor (ATF)-2 (214).

Another member of the PRR family is the NLRs. These receptors function in cellular processes that are important for immune responses to pathogens (215). Some NLRs, such as NLRP3, are essential for formation of the inflammasome, a protein involved in inflammation and apoptosis by activating host caspases (216). Interestingly, during RSV infection, signaling activated through TLR2 provides the first signal for NLRP3 expression. Once NLRP3 is translated, it forms the NLRP3/ASC inflammasome, a complex that is activated by reactive oxygen species (ROS) (217).

After RSV infects AECs it also induces NF-kB activation causing secretion of cytokines and chemokines, such as chemokine (C-C motif) ligand (CCL-5), CCL2, CXCL8, and CXCL10 (218). These cytokines have chemotactic properties in inflammatory cells and other cell types (47). Secretion of these molecules promotes the recruitment of an arsenal of immune system cells such as neutrophils, eosinophils, monocytes, macrophages, DCs, memory cells, Th1 cells, and NK cells to infected tissues (47). Secretion of TSLP from AECs contributes to an inflammatory environment in the lung. TSLP is a cytokine that plays a critical role in development of allergic asthma in AECs by functioning through the TSLP receptor (TSLPR) on myeloid DCs (219), which then triggers a second round of inflammatory cytokine secretion in RSV-infected tissues, causing lung damage (220).

Type 2 innate lymphoid cells (ILC2) and other cells of the innate immune response are recruited at the alveolarization stage of the lungs (135). After RSV infection, the pulmonary epithelium of neonates can produce large amounts of IL-33, which is associated with ILC2 accumulation during the alveolar period (221, 222). In contrast, IL-33 is not observed in lungs of adult mice in early RSV infection (135). IL-33 increases the production of ILC2 and IL-13 in lungs of neonatal mice, and impacts disease severity in RSV reinfected mice (54). However, the relationship between TSLP produced by respiratory epithelium and ILC2 proliferation/activation is not well-understood in RSV-infected neonatal mice (135).

As mentioned previously, extensive neutrophil accumulation in the lungs after RSV infection and obstruction of the small airways by excess DNA-rich mucus, produces severe RSV-LRTD (223). Although NET formation was initially thought to protect against bacteria and fungi (62), it is also now known to form in response to viral diseases including influenza, human immunodeficiency virus (HIV)-1 and poxviruses. NET formation can capture HIV-1 particles (224) and a similar protective effect is seen in mice infected with poxviruses *in vitro* (225, 226). Cortjens et al. (223) showed that NETs could capture RSV particles, in a functional form, and prevent them from infecting target epithelial cells. The same study also found marked NET formation during RSV infection *in vivo* and an accumulation of NETs in dense structures that obstruct the airways without capturing the viral antigen, indicating an unfavorable response for the host.

NET formation induced by neutrophils may be favorable for the host as a local first-line immune response against RSV. However, the intense response of these cells could worsen the pathology during RSV-LRTD (223, 227, 228). Stokes et al. (228) stated that depleting these cells induces a decrease in the process of airway inflammation and mucin expression in RSV-infected mice, which supports the fact that neutrophils may be involved in the respiratory tract tamponade during RSV-LRTD (223).

### Host Adaptative Immune Response Against RSV

#### Humoral Response

In addition to innate immune responses to RSV, infants produce antibodies to the majority of RSV proteins (229). RSV infection induces development of IgM, IgA, and IgG antibodies in both blood and mucosa. These antibodies, mediated by the adaptive immune response to RSV, protect the host against reinfections. The primary immune response against RSV is not effective, but when a reinfection occurs, in children for example, IgG and IgA antibody levels increase significantly (230). These antibodies are usually directed against RSV F and G proteins to neutralize the virus (231). However, infants <6 months of age produce less antibody against F protein and thus mount a poor neutralizing response to RSV (229).

The primary humoral immune response against RSV is the induction of the IgM antibody, which is usually detected during the first 5–10 days of the infection and persists in the blood for 1 to 3 months (230). However, in some studies the IgM response remains detectable for at least 1 year (230). Conversely, RSV-specific IgG antibodies are detected in the majority of patients, and peak 20–30 days after the symptom onset (230). Interestingly, 1 year after the patient acquires their first RSV infection, levels of RSV-specific IgG antibodies begin to decline (229). Likewise, a decreased numbers of RSV-specific neutralizing antibodies is observed in the serum of elderly adults that correlate with greater risk of developing symptomatic RSV infection (73).

Some studies showed that production of specific anti-RSV antibodies regulate the T-cell response to RSV (232). Responses mediated by T cells and antibodies are interdependent. During RSV infection of human peripheral blood mononuclear cells (PBMC), the balance between the number of CD4+ and CD8+ T cells directly depends on the relationship between neutralizing and non-neutralizing antibodies *in vitro* (233). In *in vitro* studies, RSV infection significantly increased the proinflammatory effects of substance P, a neuropeptide with bronchoconstrictor effects in animal models, by up-regulating the expression and density of its specific NK1 receptor in target cells (234). RSV not only affects substance P, but also induces specific cellular immune adaptive responses, including lymphocyte transformations, and responses mediated by cytotoxic T cells and by antibodies dependent on cytotoxic T cells (235).

#### Cellular Response

The cellular immune response to RSV infection is balanced between Th-1 and Th-2 responses (229). The Th-1 response induces IFN-γ release from CD8+ and CD4+ T lymphocytes, neutralizing antibodies, and production of mucosal IgA antibodies. While the Th-2 response induces IL4 secretion of CD4+ T cells, eosinophilia, and high levels of IgE antibody (236–238). Host factors and RSV antigens determine the balance between Th-1 and Th-2 responses (229). For example, the RSV F protein induces a Th-1 response while the G protein stimulates a Th-2 response (239). Interestingly, infants <3 months of age have higher Th-2 cytokines in nasal secretions than do older children (240). A third subset of effector T helper cells that produce IL-17 (Th17 cells), are also involved in RSV infection (241). A study of plasma cytokine profiles in infants infected with RSV (6 months or less) found that patients with a moderate response to the virus had higher IL-17 plasma levels than those with an elevated response to RSV (241). Further, IFN-γ and TNF-α levels were lower in RSV-infected infants than in controls. In contrast, another study examined tracheal aspirates and reported higher IL-6 and IL-17 levels in critically ill ventilated infants upon RSV infection than in healthy infants (242). The role of IL-17 in the respiratory tract remains unclear and further studies are needed to explain why in some cases, but not all, higher IL-17 levels are associated with improvements in RSV-infected infants. It is possible that the immature immune system in newborns presents an altered Th1 response (243) that allows favorable outcomes. Unlike adults, DCs from umbilical cord blood of RSV-infected newborns, produced IL-17 when they were co-cultured with T lymphocytes (244). In the same study, DCs of RSV-infected children were found to produce TGF-β, a cytokine that promotes differentiation of Th17 lymphocytes (244). In another study, human bronchial epithelial cells chronically infected with the long strain RSV A2, promoted differentiation of naïve T lymphocytes to Th2 and Th17 lymphocytes, but not to Th1 lymphocytes (245). Together, these studies indicate that in addition to Th1 and Th2 responses, the Th17 response also occurs in RSV infection, and suggests that the Th17 response is beneficial in some cases of RSV infection. However, Th17 responses have also been linked to respiratory tract pathology during severe asthma dominated by neutrophils. Therefore, more studies and research are needed to identify the consequences of the IL-17 production, its benefits and damages (244).

**Figure 2:**
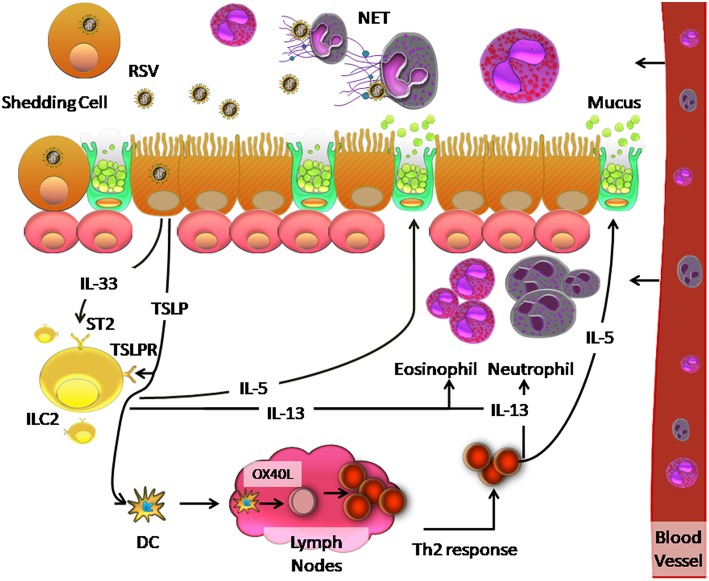
Model of the innate immune response and Th2 response against RSV infection in the human respiratory tract. RSV infection of ciliated cells induces the release of TSLP and IL-33, which are recognized by TSLP and ST2 receptors on ILC2 cells, respectively. This recognition causes the release of IL-5 and IL-13, which induce increased mucus secretion by the goblet cells and recruit neutrophils and eosinophils in the lung, respectively. Neutrophils can generate a network of DNA called NETs, which can trap and eliminate different pathogens. On the other hand, TSLP induces OX40L expression on the surface of dendritic cells, which causes these cells to migrate to the lymph nodes where they interact with naïve CD4+ T cells by binding of OX40 on these cells. The resulting Th2 immune response causes inflammation in the lung.

The inflammatory process generated upon RSV infection, may be influenced by the ability of RSV to induce TSLP production, which polarizes the cellular response to RSV. Qiao et al. (63) suggest that TSLP secretion activates mDCs in AEC caused by RSV infection, which induces polarization toward a Th2 response. This occurs because thymus- and activation- regulated chemokine (TARC/CCL17) is associated with recruitment of Th2 response cells (63). In addition, ILC2 also induces Th2-type cytokines such as IL-4, IL-5, and ILC3 via IL-17 (246) to generate a Th2 response. Activation of mDCs by TSLP allows them to migrate to draining lymph nodes, initiate an adaptive response to allergies, and promote differentiation of naïve CD4+ T cells to Th2 phenotypes, which secrete IL-5 and IL-13 (246). Upon IL-13 induction, eosinophils and neutrophils are recruited to the lung and IL-5 secretion stimulates mucus production by ciliated airway cells (246). A proposed model of this orchestrated cellular immune response to RSV is shown in Figure 2.

During RSV LRTI, systemic T-cell lymphopenia can occur due to reduced numbers of CD8+, CD4+, and CD3+ T cells, compared to those present during convalescence and in uninfected infants (241, 247, 248). In circulating T cells, CD119 expression is not increased, suggesting that these cells are not activated. Also, increased expression of cytotoxic T-lymphocyte antigen (CTLA) 4, a negative regulator of T-cell activation, is observed (249, 250). During RSV infection, T-cell lymphopenia is more pronounced in younger patients (251).

Other studies have considered that adaptive immune responses mediated by T cells and by proinflammatory cytokines, play a major role in RSV pathogenesis in children (28, 252), with little evidence that this occurs in adult patients. Specifically, the severity of RSV disease in adults and the elderly is attributed, among other factors, to low levels of specific serum antibodies against RSV (253, 254). Walsh et al. (255) suggests that the functional capacity of CD8+ T cells in adults is lost over the years (255), which contributes to increased disease severity, while several other studies suggest that immune responses mediated by these cells lose specificity instead (256, 257). Infants under 21 days of age have low numbers of CD8+ and CD4+ T lymphocytes because their adaptive immune system is not yet fully developed. Likewise, adults over 65 years of age, have fewer lymphocytes than do adults under 50 years of age, because lymphocyte numbers decrease with age. These results suggest that CD8+ and CD4+ T lymphocytes play important roles in controlling RSV infection in the host.

RSV pathogenesis in the respiratory system is caused by virulence factors, such as NS2 that provoke cell rounding, detachment of ciliated cells from the airway epithelium and contribute to airway obstruction. In contrast, host factors including age can determine immune system status and thus influence the immune response to efficiently clear RSV with minimal inflammation of lung tissue. Indeed, RSV infection in hosts with immature immune and respiratory systems produces more severe disease. Specifically, RSV infection in the airway epithelium induces TSLP and IL-33 production, which elevates the number of ILC2 at this age. In turn, these cells secrete IL-5 and IL-13, causing mucus secretion by goblet cells and recruiting proinflammatory immune cells, such as eosinophils and neutrophils. TSLP is also known to polarize DCs toward a Th2 immune response, and to stimulate proinflammatory cytokines and immune cells, such as neutrophils, which simultaneously release NETs that may contribute to RSV pathogenesis in the lower airways. A better understanding of host factors that contribute to disease severity caused by RSV will help efforts to develop therapeutic tools, such as vaccines to prevent severe RSV diseases.

## Candidate Vaccines Against RSV

After failure of the first RSV vaccine that used whole RSV inactivated with formalin in the 1960's (258), there have been significant efforts to develop therapies and new vaccine candidates using different approaches. Currently, passive immunity with palivizumab is the mainstay of RSV prophylaxis (259). This therapy, which uses a specific-RSV monoclonal antibody developed by *MedImmune*, was licensed in 1998 and is the only prophylactic tool that is effective and safe to prevent RSV infection (260, 261). It is also used to prevent serious RSV infections in high-risk children, including children born at <35 weeks gestational age (wGA) and <6 months at the onset of the RSV season, children <2 years of age who required treatment for borderline personality disorder within the last 6 months, and children <2 years of age with hemodynamically significant congenital heart disease CHD (HS-CHD) (259). However, more evidence of its usefulness is still needed in certain high-risk populations, such as those with cystic fibrosis.

Vaccine candidates for maternal immunization have been the focus of much effort worldwide (259) because it is a permissible route for childhood prophylaxis against RSV. However, strict safety standards required for any treatment or medication in pregnant women has limited progress of these strategies. Thus, vaccine candidates based on nanoparticles and subunits have been tested in clinical trials, as they would be appropriate for use in pregnant women (259). Vector and subunits vaccines attenuated *in vivo* are considered most suitable for pediatric populations because they are safer than other strategies. The drawback of these vaccine approaches is that they may mount a less robust immune response to RSV (262). Due to the unfortunate experience with the first RSV vaccine, which caused some deaths in vaccinated children upon RSV infection (263), researchers have been very cautious in designing new vaccine candidates for infants (263).

There is consensus that pediatric populations are the main risk group, and that most efforts should focus on developing an effective and safe candidate vaccine for this group. Specifically, there is a high priority to obtain a candidate vaccine for babies in their first 6 months of life, since the risk of acquiring severe RSV disease is greater in this group (263) than in infants older than 6 months of age with a more mature immune system, which reduces their susceptibility to RSV complications (263).

Currently, 84 studies of vaccine candidates against RSV are reported in the *ClinicalTrials* database (https://ClinicalTrials.gov/). Particularly, studies based on RSV subunits are in phase II and those based on RSV particles are already in phase III trials (Table 1) (259, 264). Several promising candidate vaccines and therapies based on monoclonal antibody compounds and other strategies in pre-clinical studies are also expected to be available in coming years (259).

**Table 1:** Summary of vaccines against RSV in different clinical phases according to database.

	**Preclinical**	**Phase I**	**Phase 2**	**Phase 3**	**Market approved**
Live-Attenuated/ Chimeric	Codagenix, LID/NIAID/NIH RSV	Intravacc^P^ Delta-G RSV			
		Sanofi,^P^ LID/NIAID/NIH RSV ΔNS2/ Δ1313/I1314L			
	LID/NIAID/NIH RSV	Sanofi,^P^ LID/NIAID/NIH RSV 6120/ΔNS2/1030s			
	LID/NIAID/NIH PIV1-3/RSV	Pontificia^P^ Universidad Catolica de Chile BCG/RSV			
		SIIPL, St. Jude^P^ Hospital SeV/RSV			
	MeissaVaccines RSV	Sanofi,^P^ LID/NIAID/NIH RSV D46/NS2/N/ΔM2-2-HindIII			
Whole-Inactivated	Blue WillowBiologics RSV				
Particle-Based	AgilVax VLP	Novavax^P^ RSV F Nanoparticle	Novavax^E^ RSV FNanoparticle	Novavax^M^ RSV F Nanoparticle	
	Fraunhofer VLP				
	Georgia StateUniversity VLP				
	Icosavax VLP				
	University of*Massachusetts* VLP				
	TechnoVax VLP				
	Virometix VLP				
	Artificial CellTechnologies Peptide microparticle				
Subunit	Instituto de Salud CarlosIII RSV FProtein	Beijing Advaccine^PE^ Biotechnology RSV G Protein	Pfizer^EM^ RSV FProtein		
	University ofGeorgia RSV GProtein	Immunivaccine, VIB^E^ DPX-RSV-SH Protein			
	Sciogen RSV GProtein	NIH/NIAID/VRC^EM^ RSV F Protein			
	University ofSaskatchewan RSV FProtein	GlaxoSmithKline^EM^ RSV F Protein			
		Janssen^E^ Pharmaceutical RSV F Protein			
Nucleic Acid	CureVac RNA				
	InovioPharmaceuticals DNA				
Recombinant Vectors	BravoVax Adenovirus	Vaxart^E^ Adenovirus	Bavarian Nordic^E^ MVA		
			Janssen^PE^Pharmaceutical Adenovirus		
			GlaxoSmithKline^P^ Adenovirus		
Immuno-Prophylaxis/ Combination	Arsanis Anti-F-mAb	Merck^P^ Anti-F mAb	MedImmune,^p^Sanofi Anti-F mAb		MedImmune^P^ Synagis
	Biomedical ResearchModels DNA prime, Particleboost				
	Pontificia Universidad Catolica deChile Anti-N mAb				
	UCAB,mAbXience Anti-F mAb				

*Adequate from the PATH (formerly the Program for Appropriate Technology in Health). Target indication: p, Pediatric; M, Maternal; E,Elderly*.

## Conclusions

Taken together, the virus and host both contribute to the severity of RSV disease.

RSV pathogenesis in the respiratory system is caused by virulence factors that provoke cell rounding and detachment of ciliated cells from the airway epithelium contributing to airway obstruction. Host factors including age, malnutrition, and premature delivery influence the immune system and its ability to mount an effective response to efficiently clear RSV with minimal inflammation in lung tissue. RSV infection at an early age, when the host has immature immune and respiratory systems, can produce more severe disease. Moreover, the low abundance of RSV-specific memory CD8+ T cells, which decreases with age, in older adults, is a host factor that contributes to severe RSV disease, due to loss of T-cell functional capacity and specific response to RSV in older patients. Therefore, the elderly are more likely than younger adults to present with severe RSV disease. Identification of specific genes that influence the probability of developing severe RSV disease, especially those involved in immune signaling pathways, will be important in ongoing efforts to improve immune responses that promote more efficient clearing of RSV infection from the respiratory tract. A better understanding of host factors that contribute to RSV disease severity will help us develop more effective therapeutic tools and vaccines to prevent severe RSV diseases.

## Author Contributions

JC, AA, CS-A, and JM wrote the manuscript. AK and ML reviewed the manuscript, and ML reviewed and approved the version to be published. All authors listed have made substantial and intellectual contribution to the work.

### Conflict of Interest Statement

The authors declare that the research was conducted in the absence of any commercial or financial relationships that could be construed as a potential conflict of interest.
